# Prospects for the Development of Odour Baits to Control the Tsetse Flies *Glossina tachinoides* and *G. palpalis s.l.*

**DOI:** 10.1371/journal.pntd.0000632

**Published:** 2010-03-16

**Authors:** J. B. Rayaisse, I. Tirados, D. Kaba, S. Y. Dewhirst, J. G. Logan, A. Diarrassouba, E. Salou, M. O. Omolo, P. Solano, M. J. Lehane, J. A. Pickett, G. A. Vale, S. J. Torr, J. Esterhuizen

**Affiliations:** 1 Centre International de Recherche-Développement sur l'Elevage en zone Subhumide (CIRDES), Bobo-Dioulasso, Burkina Faso; 2 Natural Resource Institute, University of Greenwich, Chatham, Kent, United Kingdom; 3 Institut Pierre Richet, Abidjan, Côte d'Ivoire; 4 Rothamsted Research, Harpenden, United Kingdom; 5 International Center for Insect Physiology and Ecology, Nairobi, Kenya; 6 Masinde Muliro University of Science & Technology, Kakamega, Kenya; 7 IRD, UMR 177 IRD/CIRAD, CIRDES, Bobo-Dioulasso, Burkina Faso; 8 Liverpool School of Tropical Medicine, United Kingdom; Foundation for Innovative New Diagnostics (FIND), Switzerland

## Abstract

Field studies were done of the responses of *Glossina palpalis palpalis* in Côte d'Ivoire, and *G. p. gambiensis* and *G. tachinoides* in Burkina Faso, to odours from humans, cattle and pigs. Responses were measured either by baiting (1.) biconical traps or (2.) electrocuting black targets with natural host odours. The catch of *G. tachinoides* from traps was significantly enhanced (∼5×) by odour from cattle but not humans. In contrast, catches from electric targets showed inconsistent results. For *G. p. gambiensis* both human and cattle odour increased (>2×) the trap catch significantly but not the catch from electric targets. For *G. p. palpalis*, odours from pigs and humans increased (∼5×) the numbers of tsetse attracted to the vicinity of the odour source but had little effect on landing or trap-entry. For *G. tachinoides* a blend of POCA (P = 3-n-propylphenol; O = 1-octen-3-ol; C = 4-methylphenol; A = acetone) alone or synthetic cattle odour (acetone, 1-octen-3-ol, 4-methylphenol and 3-*n*-propylphenol with carbon dioxide) consistently caught more tsetse than natural cattle odour. For *G. p. gambiensis*, POCA consistently increased catches from both traps and targets. For *G. p. palpalis*, doses of carbon dioxide similar to those produced by a host resulted in similar increases in attraction. Baiting traps with super-normal (∼500 mg/h) doses of acetone also consistently produced significant but slight (∼1.6×) increases in catches of male flies. The results suggest that odour-baited traps and insecticide-treated targets could assist the AU-Pan African Tsetse and Trypanosomiasis Eradication Campaign (PATTEC) in its current efforts to monitor and control Palpalis group tsetse in West Africa. For all three species, only ∼50% of the flies attracted to the vicinity of the trap were actually caught by it, suggesting that better traps might be developed by an analysis of the visual responses and identification of any semiochemicals involved in short-range interaction.

## Author Summary

Sleeping sickness, otherwise known as Human African Trypanosomiasis, continues to be a serious threat to human health. This disease, which is transmitted by tsetse flies, normally afflicts poor and isolated communities. No vaccines or prophylactic drugs are available to prevent the disease, which, once it has been contracted, is treated with curative drugs that often prove ineffective because of emerging disease resistance in the trypanosomes. These drugs can often have unpleasant and sometimes fatal side effects. Prospects for development of effective vaccines or prophylactic drugs are poor. Killing the tsetse fly vector remains the only method of preventing disease transmission. This can be done at either a local level or regionally. However, a major problem is the cost and logistical difficulty of implementing fly control programmes. To overcome this, we are trying to develop cost-effective insecticide-treated targets by identifying chemicals that will increase the numbers of tsetse that will be lured to a target and killed. Here we show that *G. tachinoides* is significantly attracted to cow odour, *G. p. gambiensis* to both cow and human odour, and *G. p. palpalis* to odours from pigs and humans. This opens the way for further work to identify the attractants present in these natural odours that can then be simply and cheaply incorporated into targets to reduce the cost of control.

## Introduction

Tsetse flies (Diptera: Glossinidae) infest ∼10 million km^2^ of sub-Saharan Africa where they transmit trypanosomes which cause Human African Trypanosomiasis (HAT; also known as sleeping sickness) and African Animal Trypanosomiasis (AAT; also known as Nagana). This complex of diseases has an important impact on health and productivity in sub-Saharan Africa [1],[2]. HAT occurs in two forms; “rhodesiense” which is caused by *Trypanosoma brucei rhodesiense* and occurs in eastern and southern Africa; “gambiense” which is caused by *T. b. gambiense* and occurs in western and central Africa. Currently the latter causes ∼97% of the total number of reported cases of HAT [1] and is transmitted in West Africa by tsetse of the Palpalis group where the most dangerous species are *G. palpalis* s.l. and *G. tachinoides*.

Means of tackling HAT and AAT differ fundamentally. Control of AAT transmitted by riverine flies is funded and implemented largely by livestock keepers [3] who treat their livestock with trypanocides and insecticides and/or deploy odour-baited traps or targets to control tsetse. Control of HAT is managed and funded by intergovernmental and national agencies and, in the case of the gambiense form, relies mainly on systematic screening, treatment and follow-up of millions of human individuals across the affected region [1]. With a few local exceptions [4] vector control has generally played little role in the management of HAT over the past 80 years. Paradoxically, vector control could contribute significantly to the management of HAT. The relatively low infection rates (<0.1%) and long incubation period (∼25 days) of *T. brucei* spp. in the vector [5], compared to the *Trypanosoma* spp. of veterinary importance, means that comparable reductions in the density and life-expectancy of tsetse populations would have a relatively greater effect on HAT than AAT. A cost-effective method of tsetse control that could be implemented by local people would complement the efforts of agencies that support mass screening and treatment and hence improve sustainability. Analyses of the history of efforts against sleeping sickness reveal that sustainable solutions have proved elusive [6],[7]. An integrated approach, based on a combination of interventions directed at both tsetse and trypanosomes, may provide a better way forward.

Cost-effective methods of tsetse control exist for the Morsitans group tsetse that spread AAT. Insecticide-treated cattle (or other domesticated animals) are valuable where they are present in sufficient numbers and form a major part of the diet of the flies. Where that is not the case or where cattle are not the major component in the fly diet, as is true in much of the cotton belt of West Africa, insecticide treated targets can be substituted. For Morsitans group flies these can be baited with attractants which mimic the odours of the natural host and they can then be deployed at densities of just 4 targets/km^2^ to eliminate fly populations [8],[9]. However, far higher densities of traps or targets (e.g. 30–50/km^2^) are required to eliminate *G. palpalis* spp. [10],[11],[12],[13]. One reason such high densities of artificial baits are required is that attractants effective against the major tsetse fly vectors of *T. brucei gambiense* in West Africa have not been identified so far.

Ironically, the genesis of modern methods of tsetse control using artificial baits started with the work of Claude Laveissiere and others, working in the HAT foci of Côte d'Ivoire during the 1970s. Their work showed that traps and targets could be used to control HAT [14] but efforts to improve the performance of traps by baiting them with the attractants effective for Morsitans group flies were not successful (C. Laveissière pers. com). Work on *G. tachinoides* in Burkina Faso [15] showed that natural odour from a human, a pig or a cow increased the catch 1.2×. In subsequent studies, they demonstrated that a combination of 3-methylphenol and octenol doubled the catch of this species of tsetse [15],[16]). In the only study of *G. p. palpalis* [17], baiting traps with acetone or octenol, both components of cattle odour, doubled the catch of tsetse. To date however, there has not been a comprehensive analysis of the olfactory responses of the Palpalis group species that spread HAT in West Africa. Accordingly, this paper reports the results of studies of the behavioural responses of *G. p. palpalis* in Côte d'Ivoire and *G. p. gambiensis* and *G. tachinoides* in Burkina Faso to host odours. These studies aimed to assess the responses of these three species to (i) whole natural odour from pigs, humans and cattle and (ii) synthetic host odours known to be effective against other species of tsetse. Identifying attractants effective for these three species would be particularly timely since the African Union is currently initiating a major tsetse control operation in West Africa under the auspices of its Pan African Tsetse and Trypanosomosis Eradication Campaign (PATTEC).

## Materials and Methods

### Study sites

*G. p. palpalis*. During the first field season studies were carried out between February and April 2008, when the rainy season begins, at sites near Bingerville (∼05.35° N, 3.82°W), ∼25 km East of Abidjan. In the second season studies took place between December 2008 and March 2009 (the dry season) at Azaguié (05.67° N, 04.11° W), ∼45 km north of Abidjan. Annual rainfall is about 1400 mm. Both areas comprise a mosaic of lagoons, farms where tree crops such as banana, coffee, cocoa, rubber and oil palm are cultivated and the remnants of dense linear forest. Humans, pigs and cattle are present at both sites but wild mammalian hosts are scarce. *G. p. palpalis* is the only species of tsetse present at these sites.

*G. tachinoides*. Studies were undertaken along the Comoe river at Folonzo (∼09° 54′ N, 04° 36′W) in the Comoe province of southern Burkina Faso. The area receives an annual rainfall of ∼1100mm. Studies took place in the dry season between March to June 2007 and January to May 2008. In general terms fly numbers were highest in the early parts of the dry season. The study site is in a protected area, and the habitat is Sudanese gallery forest. There are several game species in relatively low abundance in the research area, including warthogs (*Phacochaerus aethiopicus*), hippopotamus (*Hippopotamus amphibus*), monitor lizards (*Varanus niloticus*), hartebeest (*Alcelaphus buselaphus*), buffalo (*Syncerus cafer*), Buffon kob (*Kobus kob*), bushbuck (*Tragelaphus scriptus*), waterbuck (*Kobus ellipsiprymnus*) and various species of monkey, snake and crocodile.

*G. p. gambiensis*. Studies were performed at the same time and sites as for *G. tachinoides*, as the two species occur sympatrically along the southern Comoe river. However the Sudanese type gallery found on the Comoe is more favourable for *G. tachinoides* [18] which occurs at much higher densities than *G. p. gambiensis* [19]. Additional studies were therefore also conducted at Solenzo (∼12°14′ N, 04°23′ W), in the Banwa province of western Burkina Faso along the Mouhoun river. Climatic conditions are similar to those along the Comoe river, with an annual rainfall of 1000mm. Studies were undertaken in the dry season between April–June 2007 and January–June 2008. The habitat along the river, classed as Sudano-Guinean gallery forest [18], is favourable for the two species, and forms a narrow corridor between agricultural fields and small patches of woodland, but is heavily degraded due to expansion of agricultural fields. Host species in the area include humans, cattle, goats and pigs.

### Natural host odours

**Figure 1:**
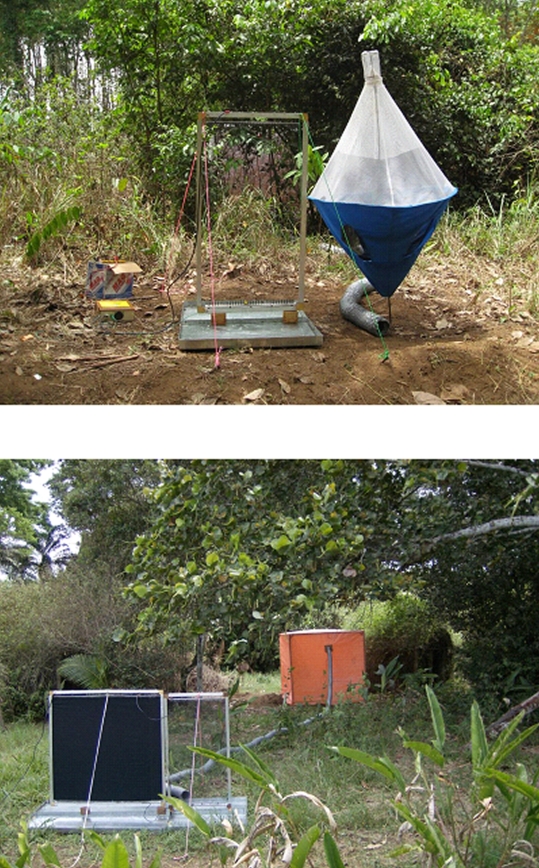
(Top) Biconical trap and (Bottom) E-target with flanking E-nets. The grey pipe leading up to the trap or target carries odour-laden air from a tent, visible in the background, containing live hosts.

At each study site, local cattle, pigs or humans were used as sources of natural host odours. The baits were placed in PVC-coated tents (∼3×2×2 m) from which the air was exhausted at ∼2000 L/min using a 12 v co-axial fan connected to a flexible PVC-coated tube (0.1 m dia.) with the outlet placed at ground level, ∼15 m away from the tent, where the various catching devices were placed (Fig. 1B). Studies with Morsitans group flies suggest that the effectiveness of odours from particular host species is related to the gross weight of animals used. Accordingly, to match the weights of different host species, tents normally contained a single ox, two men, or three pigs. Given the approximate weight of the cattle (∼150 kg), humans (∼75 kg) and pigs (∼50 kg) used, the gross weight of baits within the tent was 150–200 kg unless reported otherwise. When numbers of animals/humans in the tent varied from this it is noted at the relevant point in the text. In most instances the same animals/humans were used in the tent experiments but for logistical reasons this was not always the case.

### Synthetic host odours

In some experiments, studies were made of the responses of tsetse to chemicals known to be present in cattle odour and known to attract some species of tsetse. Chemicals were dispensed following established methods [20],[21]. Synthetic cattle odour, as used in these experiments, consisted of carbon dioxide (∼1 L/min), acetone (∼5 mg/h), racemic 1-octen-3-ol (∼0.1 mg/h), 4-methylphenol (∼0.4 mg/h) and 3-*n*-propylphenol (∼0.04 mg/h). In some experiments chemicals were dispensed individually or as blends, at rates known to be effective for other species of tsetse. For these experiments the doses of 1-octen-3-ol (∼0.2 mg/h), 4-methylphenol (∼0.4 mg/h) and 3-n-propylphenol (∼0.02 mg/h) were similar to those used with synthetic cattle odour, but the dose of acetone was increased to ∼500 mg/h. In experiments where 3-methylphenol was used the release rate was ∼0.4 mg/h. POCA consisted of P = 3-n-propylphenol (∼0.02 mg/h); O = 1-octen-3-ol (∼0.2 mg/h); C = 4-methylphenol (∼0.4 mg/h); A = acetone (∼500 mg/h). Other blends and doses are as indicated in the text.

### Catching devices

#### Traps

All traps used in the experiments were biconical traps (Fig. 1A) [11],[22].

#### Electrocuting devices

The numbers of tsetse attracted to various baits was assessed using an electric net (E-net) of fine black polyester netting (Quality no. 166, Swisstulle, Nottingham, UK), 1 m tall×0.5 wide, mounted adjacent to an electric target (E-target) of black cotton cloth, 1×1 m (Fig. 1B). Each side of the E-net and E-target was covered with a grid of fine (0.2 mm diameter) copper wires, spaced 8 mm apart. A potential difference of ∼40 KVa was created between adjacent wires and tsetse that either landed on the E-target or collided with the E-net were electrocuted and fell into a tray (3 cm deep) of soapy water on which the E-net and E-target were mounted. The fine netting and electrocuting wires of the electric net are effectively invisible to tsetse [23],[24],[25] and thus the E-net catches tsetse as they fly around the target. The total catch (i.e. E-target + E-net) provided a relative measure of the numbers of tsetse that are attracted to a target. Host odours may also affect the landing responses of tsetse [20]. Accordingly, the catches from the E-target and E-net were recorded separately to distinguish those flies caught as they landed on the target from those that collided with the net.

### Traps with E-nets

Odours that increase the catch of traps may attract more tsetse to the vicinity of the trap and/or increase the proportion of flies that enter and remain within the trap. The number of flies caught by the trap expressed as a proportion of the total flies attracted to the vicinity of the trap is termed the trap's efficiency [26] – exactly how this measured is explained below. To obtain relative measures of (i) attraction and (ii) trap entry independently, experiments were performed with an E- net (0.5 m wide×1 m high) placed adjacent to the trap (Fig. 1A). Two methods were used with *G. p. gambiensis* to assess whether odours had an effect on trap entry and efficiency. In one experiment the catches from traps operated alone with or without natural host odour were compared with those from traps operated with an adjacent E-net. For this protocol, the mean daily catch from a trap alone (i.e. without an accompanying E-net) was expressed as a proportion of the total catch from a trap+flanking E-net. This proportion is termed ‘trap efficiency’. For the second method, catches from the trap and adjacent E-net were recorded separately, to distinguish flies caught in the trap from those that collided with the net and, using these data, we assessed whether odours had a significant effect on the proportion of tsetse that entered the trap – these data provide the ‘trap entry response’. Both experiments are necessary since while the second method will detect whether there is an increased propensity for flies to enter a trap, it will underestimate absolute efficiency since the flanking E-net may kill flies that would have otherwise entered the trap. For the remainder of the paper the terms ‘trap efficiency’ and ‘trap entry response’ will be used in the sense given above.

### Experimental design and analyses

All field experiments were carried out for 4 h between 08:00 h and 12:00 h local time when Palpalis group species are most active [27],[28]. In general, odour baited devices (i.e. traps, E-nets, E-targets and combinations thereof) were compared with an unbaited device over 6–12 days in a series of replicated Latin squares of days × sites × treatments. Sites were always >100 m apart.

The daily catches (*n*) were normalized and variances homogenized using a log_10_(*n*+1) transformation and subjected to analysis of variance using GLIM4 [29]. To provide a common index of the effect of odours on catches, the detransformed mean catch of tsetse from an odour-baited device was expressed as the proportion of that from an unbaited one. The value is termed the catch index; odours which, say, double or halve the catch from a trap would have catch indices of 2 and 0.5, respectively. In some experiments, the mean catch of tsetse was <1 fly/day and these results were judged to be too low for adequate statistical analysis and are therefore not presented.

Logistic regression was used to analyse the effects of odours on the proportions that landed on a target or entered a trap. The total catch (i.e. target + net, trap + net) per day from each treatment was specified as the binomial denominator and the daily catches from the target or the trap were specified as the *y*-variable. The significance of changes in deviance was assessed by either χ^2^ or, if the data were overdispersed, an *F*-test following re-scaling [30]. Unless stated otherwise, mean catches are accompanied by the standard error of the difference (SED) between means, and the term ‘significant’ denotes that the means differ at P<0.05.

### Isolation and analysis of host odours

To verify that synthetic host odours were dispensed at rates similar to those produced by natural hosts, measurements were made of the concentration of known compounds in host odours. Carbon dioxide was measured routinely using an infra-red gas analyzer (EGM-1 or EGM-4, PP Systems, Hitchin, UK). For other chemicals, samples were collected from the air exhausted from tents containing cattle (n = 3), synthetic cattle odour (n = 2) or an empty tent (n = 3), concurrent with the behaviour studies. Volatiles were entrained (1L/min^−1^) for 4 hours onto a porous polymer (Porapak Q 50/80 (50mg), Supelco, Bellefonte, USA) which was held in glass tubing (5 mm outer diameter) by two plugs of silanised glass wool. After collection, the tubes were heat sealed at the field site in glass ampoules and sent to Rothamsted Research, UK where the volatiles were eluted with redistilled diethyl ether (750 µl). Prior to analysis, the samples were stored at −22°C.

The Porapak Q was conditioned by washing with dichloromethane (4 ml) followed by one washing with redistilled diethyl ether (4 ml) and then heating at 132 °C for 2 h under a stream of purified nitrogen (90 ml min^−1^). This conditioning process was repeated three times before use.

#### Analysis of volatiles

The air entrainment extracts were analyzed by gas chromatography (GC) on both polar (DB-wax, 30 m×0.32 mm inner diameter×0.5 µm film thickness) and non-polar (HP-1, 50 m×0.32 mm inner diameter×0.5 µm film thickness) capillary columns using a HP5890 GC (Agilent Technologies, UK) fitted with a cool-on-column injector, a deactivated retention gap (1m×0.53 mm inner diameter) and a flame ionisation detector (FID). The GC oven temperature was maintained at 30 °C for 1 min after sample injection and then raised by 5 °C min^−1^ to 150 °C, then 10 °C min^−1^ to 240 °C. The carrier gas was hydrogen. Identification of volatiles within the extracts was confirmed using peak enhancement by co-injection with chemical standards. A multiple-point external standard method was used to quantify each chemical of interest in the extracts.

#### Chemicals

Chemical standards used in the laboratory were racemic 1-octen-3-ol (98%) and 4-methylphenol (99%) were obtained from Avocado, UK. 3-n-Propylphenol (98%) was obtained from Alfa Aesar, UK. Chemicals used in the field were 1-octen-3-ol from International Flavors and Fragrances (Haverhill, UK); 4- and 3-methylphenols from Sigma-Aldrich (Gillingham, UK); 3-n-propylphenol from Great Lakes Fine Chemicals (Widnes, UK) or Appropriate Applications (Berkhamsted, UK). Acetone was obtained locally (Cobel, Bobo-Dioulasso, Burkina Faso), and carbon dioxide was kindly donated by BRAKINA (Bobo-Dioulasso, Burkina Faso).

## Results

### Natural odours

#### Traps alone

Cattle odour significantly increased the catch of *G. tachinoides* ∼5× (Table 1) for both males and females whereas human odour had no significant effect. By contrast, both cattle and human odour significantly increased the catch of *G. p. gambiensis* ∼2 to 6×.

**Table 1:** Detransformed mean daily catches (transformed mean ± SED in brackets) of *G. tachinoides* and *G. p. gambiensis* from odour-baited traps expressed as a proportion (Catch Index) of that from an unbaited trap.

Species	Odour	Reps	Tsetse/day		Catch Index	
			Males	Females	Males	Females
*G.tachinoides*	Cattle	8	14.9 (1.20±0.106)	7.8 (0.95±0.146)	4.8**	5.1**
	Human	12	10.3 (1.05±0.092)	3.2 (0.62±0.118)	1.4	1.1
*G.p.gambiensis*	Cattle	10	2.6 (0.56±0.098)	3.6 (0.66±0.083)	2.8*	6.2*
	Human	10	3.2 (0.62±0.090)	2.3 (0.52±0.059)	4.4**	2.2**

Indices followed by * or ** are significant at the 0.05 or 0.01 levels respectively.

#### Electrocuting devices

Cattle, human and pig odour all increased the catches of *G. tachinoides*, but only cattle odour had a significant effect, albeit not in all experiments (Table 2). There was no evidence that males were more responsive than females and overall the catch increase with cattle odour was 1.6 times compared to 1.2 and 1.3 for human and pig odour, respectively.

**Table 2:** Detransformed mean daily catches (transformed means ± SED in brackets) of *G. tachinoides*, *G. p. gambiensis* and *G. p. palpalis* from odour-baited electrocuting devices (E-net + E-target) expressed as a proportion (Catch Index) of that from an unbaited E-net + E-target.

Odour	Site	Tsetse/day		Catch Index	
		Males	Females	Males	Females
*G. tachinoides*, *Burkina Faso*					
Human	Folonzo	45.5 (1.67±0.079)	35.7 (1.57±0.061)	1.4	1.3
		45.5 (1.67±0.079)	56.8 (1.76±0.061)	1.2	1.1
Pig		41.4 (1.63±0.103)	35.7 (1.57±0.089)	1.3	1.6
Cattle		45.5 (1.67±0.079)	56.8 (1.76±0.061)	1.9**	1.3
		41.4 (1.63±0.103)	35.7 (1.57±0.089)	1.7	2.1*
		30.2 (1.49±0.098)	25.4 (1.42±0.088)	1.8*	2.4*
		47.2 (1.68±0.082)	83.3 (1.93±0.076)	1.1	1.2
CO2 (1 L/min)		44.2 (1.65±0.098)	42.7 (1.64±0.088)	1.5	1.7*
Syn. cattle		41.4 (1.63±0.103)	35.7 (1.57±0.089)	3.7***	4.1***
Human	Folonzo	3.7 (0.67±0.099)	2.1 (0.49±0.102)	1.5	2.0
	Solenzo	6.4 (0.87±0.113)	8.0 (0.95±0.094)	1.4	1.4
		2.5 (0.55±0.099)	5.2 (0.79±0.088)	1.1	1.4
Pig	Folonzo	3.0 (0.60±0.140)	1.9 (0.46±0.159)	1.0	0.9
		3.7 (0.67±0.099)	2.1 (0.49±0.102)	1.5	1.3
	Solenzo	6.4 (0.87±0.113)	8.0 (0.95±0.094)	0.9	0.9
		2.5 (0.55±0.099)	5.2 (0.79±0.088)	1.8	1.1
Cattle	Folonzo	3.7 (0.67±0.099)	2.1 (0.49±0.102)	1.0	2.4*
		3.0 (0.60±0.140)	1.9 (0.46±0.159)	1.3	2.2
		2.0 (0.48±0.160)	1.4 (0.39±0.138)	2.9	2.5
	Solenzo	6.4 (0.87±0.113)	8.0 (0.95±0.094)	1.0	1.2
		2.5 (0.55±0.099)	5.2 (0.79±0.088)	1.1	1.2
CO2 (1 L/min)	Folonzo	1.2 (0.35±0.160)	1.0 (0.31±0.138)	1.8	1.8
POCA		3.0 (0.60±0.140)	1.9 (0.46±0.159)	1.5	2.5
*G. p. palpalis: Côte d'Ivoire*					
Human	Bingerville	6.3 (0.87±0.094)	4.8 (0.76±0.117)	1.2	1.2
	Azaguié	5.2 (0.79±0.092)	6.7 (0.89±0.095)	2.0	1.5
Humans (×5)	Azaguié	2.3 (0.52±0.082)	4.0 (0.70±0.115)	5.0***	1.5
Pig	Bingerville	5.6 (0.82±0.118)	3.4 (0.65±0.119)	2.8*	2.7
	Azaguié	3.7 (0.67±0.092)	8.0 (0.96±0.095)	1.4	1.8
Pigs (×5)	Azaguié	1.9 (0.46±0.082)	3.5 (0.65±0.115)	4.0**	1.3
Cattle	Bingerville	4.5 (0.74±0.108)	4.9 (0.77±0.173)	1.0	2.4
	Azaguié	4.4 (0.73±0.092)	6.1 (0.85±0.095)	1.7	1.4
CO2 (1L/min)	Bingerville	9.0 (1.00±0.081)	13.5 (1.16±0.104)	1.4	1.8*
CO2 (2 L/min)	Azaguié	2.1 (0.50±0.082)	4.3 (0.72±0.115)	4.6***	1.6
CO2 (2 L/min)	Azaguié	3.7 (0.67±0.077)	5.7 (0.83±0.095)	4.3***	3.9**
CO2 (2 L/min)†	Azaguié	2.4 (0.54±0.077)	4.5 (0.74±0.095)	2.8**	3.1**

All means based on 8 or 12 (Azaguié only) replicates. Indices followed by *, ** or *** are significant at the 0.05, 0.01 or 0.001 levels respectively. Cattle, human and pig odours obtained from a single ox or bull, two humans or three pigs unless indicated otherwise. Syn. Cattle = synthetic cattle odour (carbon dioxide (2 L/min), acetone (∼500 mg/h), 1-octen-3-ol (∼0.5 mg/h), 4-methylphenol (∼1 mg/h), 3-methylphenol (∼1 mg/h) and 3*n*-propylphenol (∼0.1 mg/h).

**†:** Carbon dioxide (CO2) dispensed outside. For all other experiments, carbon dioxide was dispensed inside a tent.

For *G. p. gambiensis*, looking at combined catches for males and females, odours from cattle, human and pig increased catches using electrocuting devices 1.5, 1.4 and 1.1× respectively but the mean catches were not significantly different from the control (Table 2). This is in contrast to traps where both cattle and human odour give statistically significant increases in trap capture (Table 1). Thus while host odours consistently increased the catch from electrocuting devices, there is no compelling evidence that host odours increase the numbers of *G. p. gambiensis* attracted to an odour source. If there is no increase in the number of flies attracted by the odour then a possible explanation for the differences in catches between traps and targets may be that the presence of odour increases trap efficiency.

For *G. p. palpalis* (Table 2), the results show that odours from five but not two humans and from both three and five pigs significantly increased the catch from electrocuting devices for male *G. p. palpalis*. While there were increases in female catch index for every host experiment performed none of these was statistically significant (Table 2).

### Synthetic odours

#### Synthetic cattle odour

The significant effects of host odours might be due to carbon dioxide and/or chemicals previously shown to be effective for some species of tsetse or additional chemical components (yet to be identified). Measurements of carbon dioxide produced by natural hosts in Burkina Faso and Côte d'Ivoire showed that the mean release rates were 1.3 L/min (range: 0.9–2.2 L/min, *n* = 26) for an individual ox, 1.7 L/min (range: 0.4–3.2 L/min; *n* = 25) for three humans and 1.7 L/min (range: 0.7–3.6 L/min; n = 9) for three pigs. Accordingly, we assessed the responses of tsetse to doses of carbon dioxide alone, or in combination with 3-n-propylphenol, 1-octen-3-ol, 4-methylphenol and acetone which have been identified previously in natural cattle odour. These chemicals were dispensed at doses similar to those produced by a single ox [31],[32]. The results show that for *G. tachinoides* carbon dioxide significantly increased the catch of tsetse from electrocuting devices 1.7× (P<0.05 for females only) (Table 2) but not from traps (Table 3). Synthetic cattle odour increased catches ∼4× from electrocuting devices (P<0.001 for males and females) (Table 2) and POCA significantly increases catches from traps (Tables 3 and 4). Measurements of the concentration of compounds in the synthetic and natural host odours show that the levels of 1-octen-3-ol and phenols were greater in the synthetic cattle odour than the natural (Table 5). These results suggest that for *G. tachinoides*, the response to cattle odour might be explained by the combination of carbon dioxide, 1-octen-3-ol and phenols, and that the greater response to the synthetic cattle odour might be due to the higher doses of 1-octen-3-ol and phenols it contains.

**Table 3:** Detransformed mean daily catches (transformed means and SED in brackets) (trap plus flanking E-net and trap-entry responses (%) of tsetse for devices baited with natural and synthetic host odours.

Odour	Mean daily catch		Trap entry-response±SE (%)	
	Males/day	Females/day	Males	Females
*Experiment 1. G. tachinoides (Folonzo, Burkina Faso)*				
CO2	57.1 (1.76)	97.2 (1.99)	38±3.9	12±3.1
POCA	92.1 (1.97)*	128.7 (2.11)	30±3.5	11±2.8
Cattle	52.3 (1.73)	102.3 (2.01)	38±3.0	16±2.7
None	47.2 (1.68)	83.3 (1.93)	33±3.8	11±2.7
SED	(0.082)	(0.076)		
Replicates	8			
*Experiment 2. G.p. gambiensis (Solenzo, Burkina Faso)*				
Cattle	2.8 (0.58)	6.3 (0.86)	9±6.4)	11±4.6
Human	2.9 (0.59)	7.1 (0.91)	15±7.2	14±4.5
Pig	4.5 (0.74)	5.9 (0.84)	9±5.5	12±5.3
None	2.5 (0.55)	5.2 (0.79)	15±8.2	8±4.5
SED	(0.099)	(0.088)		
Replicates	8			
*Experiment 3. G.p. gambiensis (Solenzo, Burkina Faso)*				
POCA	13.0 (1.10)	10.5 (1.02)	35±4.1	29±4.9
None	8.0 (0.90)	8.2 (0.91)	27±5.1	22±5.8
SED	(0.101)	(0.063)		
Replicates	10			
*Experiment 4. G.p. palpalis (Bingerville, Côte d'Ivoire)*				
Pig	3.9 (0.69)	4.5 (0.74)	8±6.2	19±7.0
None	3.5 (0.66)	4.1 (0.71)	16±8.4	26±8.4
SED	(0.138)	(1.116)		
Replicates	8			
*Experiment 5. G.p. palpalis (Bingerville, Côte d'Ivoire)*				
Human	4.8 (0.77)	5.0 (0.78)	14±6.3	8±3.2
None	5.3 (0.80)	5.6 (0.82)	27±6.7	15±4.0
SED	(0.115)	(0.109)		
Replicates	8			

Catches accompanied by * differ from the unbaited control trap at the 0.05 level of probability. Catches of the unbaited devices are accompanied by their respective transformed means±SED shown in brackets.

**Table 4:** Detransformed mean daily catch (transformed means±SED in brackets) of *G. tachinoides* from traps baited with synthetic host odours.

Odours					Reps.	Tsetse/day		Catch index	
A	O	4M	3nP	3M		Males	Females	Males	Females
X	X	X	X	X	12	13.3 (1.16±0.198)	5.5 (0.82±0.209)	2.3	3.0
X	X	X	X		12	15.2 (1.21±0.198)	8.8 (0.99±0.198)	2.6	4.8
X	X	X	X		8	7.7 (0.94±0.191)	8.0 (0.96±0.191)	5.8*	14.2**
X	X	X	X		8	3.8 (0.68±0.194)	5.9 (0.84±0.149)	5.4	7.4**
X	X	X	X		3	9.7 (1.03±0.276)	9.8 (1.03±0.161)	16.6	2.8
X	X		X	X	12	11.2 (1.16±0.198)	6.6 (0.88±0.198)	1.9	3.6
	X	X	X		8	1.7 (0.44±0.194)	5.6 (0.82±0.149)	2.4	7.0**
	X	X	X		3	5.9 (0.84±0.276)	7.3 (0.92±0.161)	10.1	2.1
	X	X	X		12	26.2 (1.43±0.194)	14.7 (1.20±0.183)	2.8*	3.2
X					8	4.0 (0.70±0.194)	6.6 (0.88±0.149)	5.6	8.4**
X					12	21.2 (1.35±0.194)	15.7 (1.22±0.183)	2.2	3.5

Catch Index is the mean catch of an odour-baited trap expressed as a proportion of that from an unbaited trap. Asterisks indicate that the Catch Index differs from unity at the *P*<0.05 (*) or *P*<0.01 (**) levels of significance. Key to odours: A = acetone. O = 1-octen-3-ol; 3-n-P = 3-n-propylphenol; 4-M = 4-methylphenol; 3-M = 3-methylphenol. Shaded cells indicate chemicals used in each experiment.

**Table 5:** Release rates of chemicals from natural and synthetic odour sources.

	Release Rates (mean µg h^−1^ ± S.E.)		
	1-Octen-3-ol	4-Methylphenol	3-n-Propylphenol
Bull (Folonzo)	30.9±0.4	55±0.7	22.3±2.6
Bull (Solenzo)	30.5±0.2	55.5±1.2	16.5±0.1
Synthetic cattle odour	129±6	332±11	66±2

The results for *G. p. gambiensis* (Table 2) show that carbon dioxide and POCA both increased the catches from electrocuting devices but this was not statistically significant.

For *G. p. palpalis* (Table 2), carbon dioxide dispensed at 1 or 2 L/min increased the electrocuting device catch of male and/or female tsetse significantly in four separate experiments (Table 2). The higher dose produced a greater increase, consistent with the responses to natural host odours. For instance, the odour from two humans increased the electrocuting device catch for male flies <2 fold whereas the odour from five humans increased it five-fold (Table 2). Similarly, carbon dioxide dispensed at 1 L/min or 2 L/min increased the catch by up to 1.8× and 4.6×, respectively. These results suggest that the response of *G. p. palpalis* to host odour might be due to carbon dioxide. Dispensing carbon dioxide within a tent – so its source concentration was comparable to that with natural host odours (∼0.1%) – or directly from a pipe connected to the cylinder so that the source concentration was 100% made no clear difference to its effect (Table 2).

Components of ox odour [31],[32] dispensed in the absence of carbon dioxide increased the catch of *G. tachinoides* and *G. p. gambiensis*. For *G. tachinoides* POCA consistently increased the trap catch, albeit the increases were not always statistically significant (Table 4). Pooling the results for the 31 replicates where a POCA-baited trap was compared with an unbaited one showed that POCA increased the catch of males four-fold, from 2.1 (0.50±0.104) males/day to 8.5 (0.98±0.104) males/day and the catch of females increased six-fold, from 1.3 (0.36±0.114) females/day to 7.5 (0.93±0.114) females/day (*P*<0.001 for difference between means for both sexes). Baiting an E-target with POCA also increased the catch from 30.2 males and 25.4 females without odour to 53.8 and 50.8, respectively, with POCA (*P*<0.05 for both sexes).

When the numbers of *G. p. gambiensis* caught were sufficient to allow robust statistical comparisons (>3 tsetse/trap/day for an unbaited trap), the blends which gave the best results were POCA and POC (i.e. POCA without acetone) (see Table 6). Pooling the results for the 78 replicates where a POCA-baited trap was compared with an unbaited one showed that POCA increased the catch significantly. The catch increased 2.2× for males, from 2.3 (0.51±0.050) males/day to 5.1 (0.78±0.050) males/day and by 1.8× for females increasing from 3.7 females/day (0.67±0.063) without odour to 6.1 (0.85±0.063) females/day with POCA. Baiting an E-target with POCA also increased the detransformed mean daily catch of males from 6.9 to 13.3 (P<0.001 for difference between means) and of females from 8.0 to 13.3 (P<0.01).

**Table 6:** Detransformed mean daily catch (transformed means±SED in brackets) of *G. p. gambiensis* from traps baited with synthetic host odours.

A	O	4M	3nP	Reps.	Tsetse/day		Catch index	
					Males	Females	Males	Females
X	X	X	X	8	8.8 (0.99±0.086)	2.5 (0.55±0.128)	4.7***	1.9
X	X	X	X	10	5.9 (0.84±0.130)	7.9 (0.95±0.140)	1.9	1.4
X	X	X	X	12	1.6 (0.41±0.107)	3.0 (0.60±0.155)	0.9	0.8
X	X	X	X	16	6.4 (0.87±0.099)	5.8 (0.78±0.101)	2.9**	1.2
X	X	X	X	12	4.8 (0.76±0.154)	9.4 (1.02±0.110)	1.9	2.5*
X	X	X	X	20	5.8 (0.84±0.080)	8.9 (0.99±0.067)	2.5***	2.5***
	X	X	X	12	2.4 (0.53±0.107)	2.9 (0.60±0.155)	1.4	0.8
	X	X	X	12	8.5 (0.98±0.154)	8.0 (0.96±0.110)	3.4*	2.2*
	X	X		12	1.8 (0.44±0.107)	3.5 (0.66±0.155)	1.1	1.0
		X	X	12	1.5 (0.39±0.107)	3.4 (0.65±0.155)	0.9	1.0
	X		X	12	1.8 (0.45±0.107)	3.3 (0.63±0.155)	1.1	0.9
X				12	1.3 (0.37±0.142)	1.0 (0.31±0.122)	1.3	1.6
	X			10	4.0 (0.70±0.130)	5.3 (0.80±0.140)	1.3	1.0
		X		10	3.4 (0.64±0.130)	6.5 (0.88±0.140)	1.1	1.2
			X	10	5.8 (0.83±0.130)	8.6 (0.98±0.140)	1.9	1.5

Catch Index is the mean catch of an odour-baited trap expressed as a proportion of that from an unbaited trap. Asterisks indicate that the Catch Index differs from unity at the *P*<0.05 (*) or *P*<0.01 (**) levels of significance. Key to odours: A = acetone. O = 1-octen-3-ol; 3nP = 3-n-propylphenol; 4M = 4-methylphenol. Cells in grey show the chemicals that were used in each experiment.

For *G. p. palpalis* (Table 7), blends containing acetone or 1-octen-3-ol increased the catch slightly (∼1.5×) and significantly in some experiments.

**Table 7:** Detransformed mean daily catch (transformed means±SED in brackets) of *G. p. palpalis* from traps baited with synthetic host odours.

A	O	4M	3nP	Site		Tsetse/day		Catch Index	
					Reps.	Males	Females	Males	Females
X	X	X	X	Azaguié	36	4.2 (0.72±0.052)	4.8 (0.77±0.052)	1.6*	1.3
	X	X	X	Bingerville	40	3.8 (0.68±0.050)	6.7 (0.89±0.065)	1.5*	1.5*
	X	X	X	Azaguié	36	2.5 (0.55±0.052)	3.9 (0.69±0.052)	1.0	1.0
X				Bingerville	40	4.1 (0.71±0.050)	5.3 (0.80±0.065)	1.6*	1.2
X				Azaguié	36	4.3 (0.72±0.052)	5.0 (0.78±0.052)	1.6*	1.3
	X			Bingerville	40	3.7 (0.67±0.050)	3.9 (0.69±0.065)	1.4*	0.9
	X			Azaguié	36	3.2 (0.63±0.052)	4.1 (0.71±0.052)	1.2	1.1
		X	X	Azaguié	36	2.6 (0.56±0.052)	4.2 (0.72±0.052)	1.0	1.1

Catch Index is the mean catch of an odour-baited trap expressed as a proportion of that from an unbaited trap. Asterisks indicate that the Catch Index differs from unity at the *P*<0.05 (*) level of significance. Key to odours: A = acetone. O = 1-octen-3-ol; 3nP = 3-n-propylphenol; 4M = 4-methylphenol. Cells in grey show the chemicals that were used in each experiment.

### Landing responses

**Figure 2:**
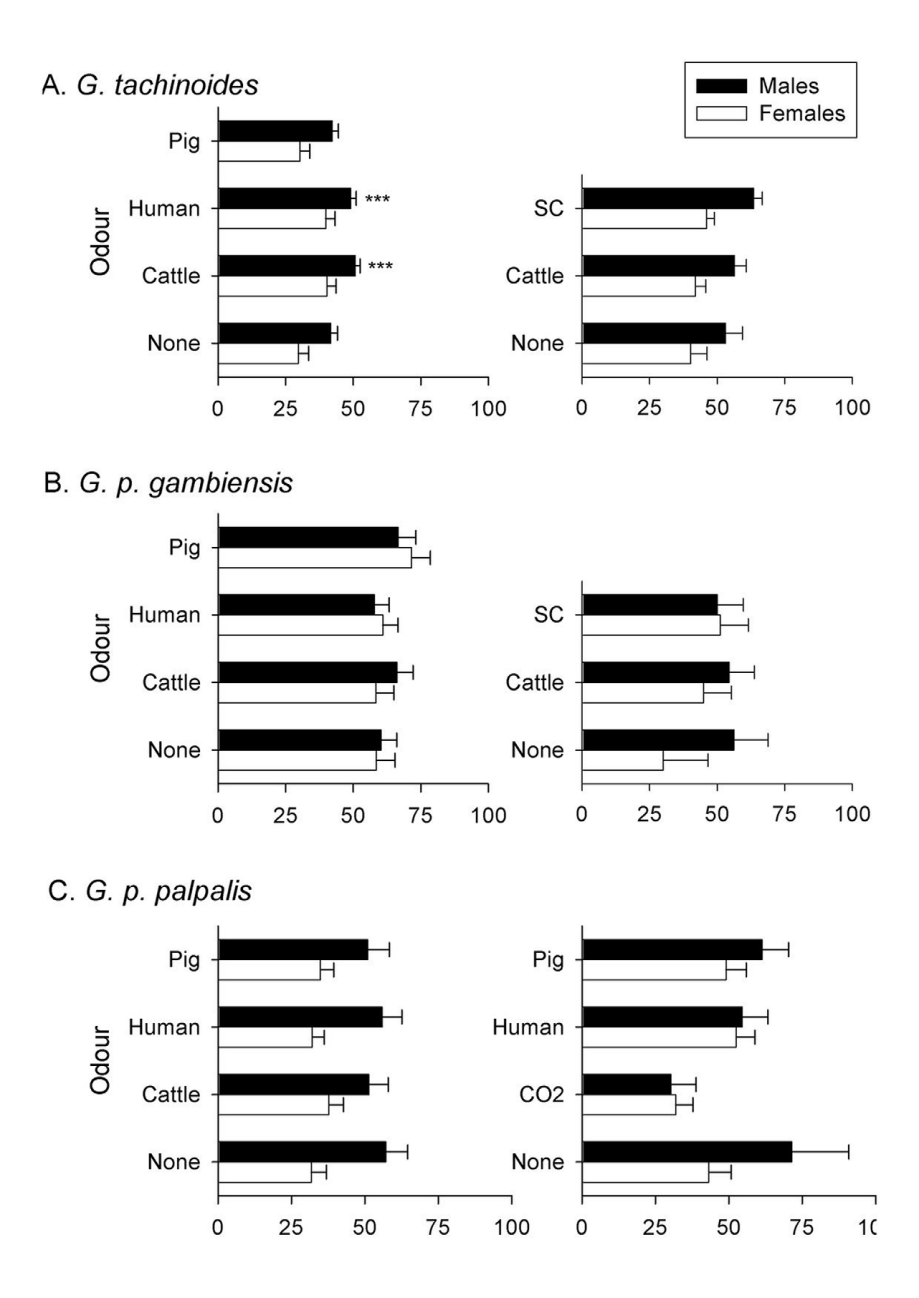
Mean percentages of *G. tachinoides*, *G. p. gambiensis* and *G. p. palpalis* landing on a cloth target baited from experiment where different natural (left-hand column) or natural vs. synthetic host odours (right-hand column) were compared. Values accompanied by * differ from the unbaited target at the 0.05 level of probability.

For the 35 separate experiments listed in Table 2 (*G. tachinoides*, 9 experiments; *G. p. gambiensis*, 14 experiments; *G. p. palpalis*, 12 experiments) we also assessed the landing responses of tsetse exposed to natural or synthetic odours. Just one, *G. tachinoides* responding to human and cattle odour, showed a significant effect (Fig. 2A). However, in other experiments, these odours did not increase the landing response of *G. tachinoides*. Similarly baiting an E-target+E-net with POCA had no significant effect on landing response. We therefore conclude that natural host odours have no clear or consistent effect on the landing responses of *G. tachinoides*, *G. p. gambiensis* or *G. p. palpalis*. Illustrative examples of the general landing responses from six experiments (i.e., two for each species) are shown in Fig. 2. For *G. tachinoides* and *G. p. palpalis*, males generally showed a stronger landing response than females but this difference was not apparent for *G. p. gambiensis*.

### Trap efficiency

Natural host odours had no significant effect on the trap entry response of *G. tachinoides*, *G. p. gambiensis* or *G. p. palpalis* (Table 3). The results show that for *G. tachinoides* there was a marked difference in the trap entry response of males and females, with 30–38% of males being caught in the trap compared to only 11–16% of females (Table 3, experiment 1). For *G. p. gambiensis*, the percentage of males and females caught in a trap was variable, ranging from 8–15% in one experiment (Table 3, experiment 2) to 22–35% in another (Table 3, experiment 3). The percentage of *G. p. palpalis* caught from a trap was generally low, ranging between 8 and 27% (Table 3, experiments 3 & 4). While host odours did not have any effect on trap entry, the results do show that the total catch (trap+flanking net) of *G. tachinoides* was increased significantly by the POCA blend (Table 3, experiment 1).

For *G. p. gambiensis*, host odours had no significant effect on total catch or trap entry response (Table 3, experiments 2 and 3). However, the data do suggest that both are increased; analysing the pooled catch of males and females did show that the catch increased significantly (from 16 to 23 tsetse/day, P<0.05) and the trap efficiencies increased for both sexes and was significant (P = 0.05) for males. The absence of any significant effects of host odours on attraction or trap efficiency for *G. p. gambiensis* may be an experimental artefact: the E-net may have killed circling flies that would have eventually entered the trap. Accordingly, we also assessed trap efficiency for *G. p. gambiensis* using the alternative protocol of comparing catches from traps with or without a flanking E-net in the presence or absence of cattle odour. The result showed that host odour had no significant effect, but placing an E-net adjacent to a trap increased the detransformed mean daily catch of both sexes significantly from 2 males and 4 females to 10 males and 13 females. Thus the catch from the trap alone was just 20–25% of that from the trap+E-net. These percentages are broadly consistent with the estimates of efficiency which are collected when using data from a trap+flanking E-net alone. Taken together, the results suggest that the trap entry response is not modulated by natural cattle odour but that total number of flies attracted to the vicinity of the trap is.

## Discussion

We report here that baiting various catching devices with natural or synthetic odours increases catches of the Palpalis group flies, *G. tachinoides*, *G. p. gambiensis* and *G. p. palpalis*. This is the first comprehensive report of odour attraction for *G. palpalis* spp. and confirms the earlier finding for *G. tachinoides* [27],[33]. This gives new promise for the use of odour-baited control devices against Palpalis group flies that transmit *gambiense* sleeping sickness in West Africa.

### Responses of *G. tachinoides* to natural and synthetic host odours

The large number of experiments done and the high numbers of flies caught provide firm evidence that *G. tachinoides* showed consistent increases in catch index of around 2× in response to natural cattle odour, confirming the previous findings [15],[27]. We obtained slightly higher increases than reported in their studies, particular with traps where cattle odour increased our catches ∼5×. Synthetic cattle odour (defined in Materials and Methods), which contains known kairomones for Morsitans group tsetse, produced greater (∼4×) increases in trap catch (Table 4) than given by the natural cattle odour (Table 1). The greater catch seen with synthetic cattle odour may be because (i) the release rate was ∼5× greater than that in the natural (determined from Table 5) or (ii) natural ox odour produces chemicals that ‘repel’ a proportion of the flies. Human and pig odours were not effective with *G. tachinoides* suggesting that the effective kairomones are found only in cattle odours or that humans and pigs are producing repellents over-riding any kairomones in their odour [34],[35].

Various combinations of acetone, 1-octen-3-ol, 3-*n*-propylphenol and 4-methylphenol are used to increase the performance of traps and insecticide-treated targets to monitor and control various Morsitans- and Fusca-group species of tsetse - see review [36]. The results confirm those of earlier studies [15],[37] showing that the POCA blend, originally developed for use against *G. pallidipes* [38] is also effective against *G. tachinoides*. Our data suggests that the incorporation in the blend of 4-methylphenol is about twice as effective as 3-methylphenol (Table 4). Our results combined with those of earlier studies [16],[37],[39], suggest a blend of POC (i.e. without acetone) may be equally effective, producing increases comparable to natural cattle odour. This point is of practical importance as the large volumes of acetone required makes its use in long running control operations particularly difficult.

### Responses of *G. palpalis* spp. to natural and synthetic host odours

Natural odours from both cattle and humans increased the catch of *G. p. gambiensis* from traps. But our extensive studies on the effect of baiting electrocuting devices with natural host odours did not show consistently significant effects. For example, for *G. p. palpalis*, natural odours from five pigs or five humans increased the catch from electrocuting devices but studies with lower numbers of hosts were ineffective. These data for traps and electrocuting devices suggest there is an interaction between odours and visual responses to the catching device. At least part of the difficulty with these studies is caused by the low densities of tsetse – a widespread problem which hampers field studies of *G. palpalis* [40]. These low densities require that very large numbers of replicates are performed for robust statistical analysis to be possible. As a consequence, the absence of statistically robust effects has perhaps led to the erroneous conclusion that *G. palpalis* spp. are unresponsive to host odours. In the present study, experiments conducted at times or places where *G. p. gambiensis* were still low but more abundant than usual did show that baiting traps with natural odours and/or synthetic blends, particularly POCA and POC significantly increased the catches. This is to our knowledge the first published report of improvement in catches using olfactory attractants for this species. Further studies of the responses of *G. palpalis* spp. are clearly needed to confirm these findings and to identify cost-effective doses and blends.

### Landing responses and trap efficiencies

Our results suggest that the three species exhibit a relatively high landing response (40–50%) which was not modulated by natural host odours. However, exhausting volatiles from the tent containing hosts through a long PVC-coated tube to the catching devices could have resulted in a reduced number and concentration of compounds with low volatility compared with those emitted by the host. Compounds with low volatility may be important cues that induce tsetse landing response and this may have caused the lack of difference in landing response to odours from different hosts and control devices. For example, Warnes [41] demonstrated that electrified targets impregnated with ox skin secretions (sebum) caught more flies than targets without sebum. The landing response of female *G. tachinoides* was lower than that of males, confirming previous observations for this species [37] and *G. p. palpalis* showed a similar trend. This was not the case for *G. p. gambiensis* where both sexes show similar responses. It should be noted that these responses are all to a single size of target. Laveissière *et al.* [40] working on *G. p. palpalis* in Côte d'Ivoire, suggested landing response of males and females varied with changing surface area with more males and less females captured as the black surface area increased.

The present results suggest that improvements could be made in the efficiency of traps for Palpalis group tsetse since only 10–30% of tsetse entered a trap. Thus while the biconical trap is the most widely used trap for control and monitoring riverine tsetse in West Africa most of the flies that are attracted to it do not enter immediately [40]. For *G. p. gambiensis*, odours were most effective when delivered with traps, suggesting the importance of visual responses as well as responses to odours. Thus analysis of visual-olfactory interactions might be the key to improving trap efficiency.

### Seeing, smelling, or both?

For both *G. p. gambiensis* and *G. tachinoides*, the overall increases in catch index, landing and entry responses were relatively small in comparison to those found with Morsitans group flies [42],[43]. In the Palpalis group flies studied here *G. tachinoides* showed higher responses to natural ox odours than *G. p. gambiensis*, and also higher responses to POCA. This is consistent with previous observations that this species' behaviour and ecology is intermediate between the savannah-dwelling Morsitans group flies and the more riverine Palpalis group species such as *G. palpalis* [4]. The smaller increases in catch indices compared to Morsitans group flies that have been observed here for *G. palpalis spp.* also apply to the other Palpalis group flies *G. fuscipes fuscipes* and *G. f. quanzensis* [44]. There is a pressing need to understand why the odours investigated here are seemingly less effective for Palpalis group tsetse. Is the poor response because they rely predominantly on visual cues or because they use odours in a different way to Morsitans group flies? It has been argued that dense vegetation could be an obstacle to the dispersion of the odour plume [45]. Indeed the riverine species that were studied here (*G. tachinoides* and *G. p. gambiensis*) live in habitats that differ considerably from the habitats where detailed studies on olfactory cues and host location in savannah tsetse have been conducted. Here their habitats are the linear forests bordering the Comoe or Mouhoun rivers. In recent years, these habitats have become highly fragmented due to human pressure. Hence in these linear and/or fragmented habitats, wind-borne odours may simply be carried to places where few tsetse are found [45]. Such an explanation would not explain the results for *G. p. palpalis* which is extensively distributed in humid and degraded forest habitats of southern Côte d'Ivoire which are not linear. Although dense vegetation may be an obstacle to the dispersal of volatile chemicals, it is unlikely that this will completely obstruct their movement through such an environment. For example, it has been demonstrated that volatile chemicals release by plants in the rhizosphere can disperse through the soil – an extremely dense environment – and are detected by neighboring plants and nematodes [46],[47],[48],[49].

Another possible explanation for the variability in the responses of *G. palpalis* spp. to host odours in the present and earlier studies (eg, [40]) may center on population structure. There is evidence that in the fragmented habitats typical of populations of *G. p. palpalis* and *G. p. gambiensis* the populations may consist of several, genetically-differentiated subunits [50],[51], and it has been suggested that these sympatric demes may respond differentially to a given stimulus [52]. Genetically-differentiated demes are associated with trypanosomes from particular host species. One possible explanation for this is that these demes feed preferentially on particular host species. Consequently, the low response to, say, cattle odour may be because only tsetse with a preference for feeding on cattle may respond strongly to cattle odours. Other studies have already reported intraspecific variations in olfactory responses for allopatric populations (e.g. *G. pallidipes* - [53]).

The present results, combined with the earlier studies [15],[44] contribute to the emerging view that Palpalis-group flies do not show the marked response to host odours exhibited by Morsitans-group tsetse. The relatively low (∼2×) increase in catch observed across a range of habitats suggests that the difference between the Palpalis- and Morsitans-groups is due to their innate host-oriented behavior rather than their particular habitats. We now need to understand better how the Palpalis-group species locate their hosts so that we have a rational basis for developing more cost-effective baits.

### Prospects for the use of olfactory attractants to control Palpalis group tsetse flies

Despite being lower than for Morsitans group flies, the increases in tsetse catches reported here promise improvements for Palpalis group tsetse control with respect to both human and animal trypanosomiases. There are immediate applications of the use of POCA to improve trapping and control. Indeed, the AU-supported PATTEC program in Burkina Faso has already begun to use this blend for pre-control entomological surveys (I Sidibe, PATTEC coordinator, Burkina Faso, pers. comm.). It is our intention to investigate in more detail the use of POCA blends and individual compounds to enhance control of Palpalis group flies. Regarding costs, we are undertaking further experiments to determine if more cost-effective blends (e.g. OC) can be used. In preliminary experiments this blend has been shown to double the catches of *G. p. palpalis* in Liberia [17] and to double catches of *G. tachinoides* in Burkina Faso [16],[37]. Present results suggest that even the relatively modest 2–4× increases in catch indicated by current results could halve the densities of targets required to control Palpalis group tsetse from the current ∼30–50 targets/km^2^ [54] with consequent significant economic and logistical benefits. Perhaps more importantly in the longer term, the present results show that there is much for improvement in the design and performance of trapping devices. In particular, there is a need to analyse the visual and olfactory responses of riverine species to their reptilian hosts, particularly monitor lizards and crocodiles which constitute an important part of the diet of *G. palpalis* and *G. tachinoides* [28],[55],[56],[57].
